# Differential Binding of Three Major Human ADAR Isoforms to Coding and Long Non-Coding Transcripts

**DOI:** 10.3390/genes8020068

**Published:** 2017-02-11

**Authors:** Josephine Galipon, Rintaro Ishii, Yutaka Suzuki, Masaru Tomita, Kumiko Ui-Tei

**Affiliations:** 1 Keio University Institute for Advanced Biosciences, Tsuruoka 997-0017, Japan; jgalipon@ttck.keio.ac.jp (J.G.); mt@sfc.keio.ac.jp (M.T.); 2 Graduate School of Frontier Sciences, The University of Tokyo, Kashiwa 277-8562, Japan; rin-go2@jcom.home.ne.jp (R.I.); ysuzuki@k.u-tokyo.ac.jp (Y.S.); 3 Graduate School of Science, The University of Tokyo, Tokyo 113-0032, Japan

**Keywords:** ADAR1-p110, ADAR1-p150, ADAR3, RIP sequence, KEGG, GO

## Abstract

RNA editing by deamination of adenosine to inosine is an evolutionarily conserved process involved in many cellular pathways, from alternative splicing to miRNA targeting. In humans, it is carried out by no less than three major adenosine deaminases acting on RNA (ADARs): ADAR1-p150, ADAR1-p110, and ADAR2. However, the first two derive from alternative splicing, so that it is currently impossible to delete ADAR1-p110 without also knocking out ADAR1-p150 expression. Furthermore, the expression levels of ADARs varies wildly among cell types, and no study has systematically explored the effect of each of these isoforms on the cell transcriptome. In this study, RNA immunoprecipitation (RIP)-sequencing on overexpressed ADAR isoforms tagged with green fluorescent protein (GFP) shows that each ADAR is associated with a specific set of differentially expressed genes, and that they each bind to distinct set of RNA targets. Our results show a good overlap with known edited transcripts, establishing RIP-seq as a valid method for the investigation of RNA editing biology.

## 1. Introduction

The post-transcriptional modification of RNA is an evolutionarily conserved mechanism and a factor contributing to transcriptome diversity. In particular, the deamination of adenosine to inosine (A-to-I editing) on double-stranded RNA (dsRNA) was first identified for its ability to regulate the activity of neurotransmitter receptors [1]. The reaction is catalyzed by members of the ADAR family of dsRNA binding proteins (adenosine deaminases acting on RNA), which comprises of three constitutive isoforms in human, labeled ADAR (ADAR1), ADARB1 (ADAR2), and ADARB2 (ADAR3). While the deaminase domain of ADAR3 is not catalytically active, it is thought to act as a competitive inhibitor of ADAR1 and ADAR2 in the brain [2]. The generation of knockout mice for *ADAR1* and *ADAR2* further revealed that ADAR1 plays an essential role in cell survival and development, as *ADAR1−/−* embryos undergo massive apoptosis in early embryogenesis from E10.5 to E11.5 [3]. *ADAR2−/−* mice on the other hand are viable, but prone to epilepsy and their lifespan is shorter than wild-type [4]. The *ADAR1* locus codes for a constitutive 110 kDa isoform (ADAR1-p110), and the 150 kDa isoform (ADAR1-p150) is generated from the same locus during the interferon response [5].

The conversion of adenosine to inosine in the coding region is biologically meaningful, as inosine behaves as guanosine by favoring base-pairing with cytidine. In earlier years, studies on A-to-I editing primarily focused on modifications within coding sequences, as editing in those regions is susceptible to generate a protein product that differs by one amino-acid from the protein predicted by the original DNA sequence, which may modulate the protein activity [1]. Furthermore, the role of inosine in the formation of tRNA wobble base pairs has been described as early as the mid-1960s [6]. RNA editing has the further potential to regulate RNA structure via the modulation of base-pairing strength, which means it can virtually influence any cellular process requiring an interaction with RNA. While adenosine (A) typically binds to uridine (U) forming A:U pairs, inosine (I) forms a wobble interaction with cytosine (C), meaning that ADARs have the potential of either destabilizing (A:U to I/U) or stabilizing (A/C to I:C) dsRNA. This dual ability positions them as potential core players in the regulation of RNA activity. Meanwhile, the advent of RNA deep sequencing technologies (RNA-seq) has revealed that the majority of editing events occur on non-coding RNA [7]. However, there is presently no study that addresses the binding target preference of every ADAR active isoform, due to a lack of antibodies specific enough to distinguish ADAR1-p110 from ADAR1-p150 [8]. The transcription of ADAR1-p150 and ADAR1-p110 initiates from two promoters at the same gene locus: one interferon (IFN)-inducible promoter initiates the transcription of ADAR1-p150, while another constitutive promoter drives ADAR1-p110 [9]. Alternative splicing of exon 1 results in two sizes of ADAR1 proteins, approximately 150 kilodaltons (kDa) for IFN-inducible ADAR1-p150 and 110 kDa for the constitutively expressed ADAR1-p110. This makes it technically impossible to knock-out ADAR1-p110 specifically without knocking out ADAR1-p150 expression. Furthermore, popular methods for detecting genome-wide binding to RNA typically use UV crosslinking, which not only is inefficient in stabilizing interactions with perfect double-stranded RNAs [10], but prevents downstream analysis of editing sites due to the introduction of sequencing errors at the sites of crosslinking. Here, we present an RNA immunoprecipitation deep sequencing (RIP-seq) strategy that identifies targets for all known catalytically active ADAR isoforms: ADAR1-p150, ADAR1-p110, and ADAR2. We analyzed the effect of overexpressing green fluorescent protein (GFP)-tagged ADAR isoforms on the expression levels of size-selected long RNAs (>200 nt) in HeLa cells, and revealed ADAR isoform-specific binding target preferences.

## 2. Materials and Methods 

### 2.1. Plasmid Construction

Total RNA was isolated from HeLa cells and cDNA was prepared by reverse transcription using random hexamers according to the manufacturer’s protocol (Transcriptor High Fidelity cDNA Synthesis Kit, Roche Molecular Systems, Pleasanton, CA, USA). The genomic sequences for ADAR1-p150 (NM_001111), ADAR1-p110 (NM_001193495), and ADAR2 (NM_001112) were amplified by RT-PCR using the following primers (ADAR1-p150-F and ADAR1-R primers for ADAR1-p150, ADAR1-p110-F and ADAR1-R for ADAR1-p110, and ADAR2-F and ADAR2-R for ADAR2), each containing a restriction enzyme cleavage site (lower case letters):

ADAR1-p150-F: 5’-AAAGGGaagcttATGAATCCGCGGCAGGGGTATTCC-3’ (*Hind*III),
ADAR1-p110-F: 5’-AAAGGGaagcttATGGCCGAGATCAAGGAGAAAATC-3’ (*Hind*III),
ADAR1-R: 5’-AAAGGGtctagaCTATACTGGGCAGAGATAAAAGTTC-3’ (*Xba*I),
ADAR2-F: 5’-AAAGGGgaattcATGGATATAGAAGATGAAGAAAACATG-3’ (*EcoR*I),
ADAR2-R: 5’-AAAAGGAAAAgcggccgcTCAGGGCGTGAGTGAGAACTGGTC-3’ (*Not*I).

The amplified fragments were each digested with the appropriate mixture of restriction enzymes (ADAR1-p150: *Hind*III+*Xba*I, ADAR1-p110: *Hind*III+*Xba*I, ADAR2: *EcoR*I+*Not*I). The pcDNA3.1(+) vector carrying pm-GFP-TNRC6A [11] was digested with *Hind*III+*Xba*I to remove the TNRC6A construct, and the digested ADAR1-p150 and -p110 fragments were inserted into pcDNA3.1(+) by ligation. The ADAR2 fragment was inserted into pcDNA3.1(+) after digestion with *EcoR*I+*Not*I. 

### 2.2. Cell Culture and Transfection

HeLa cells (3 × 10^6^) were inoculated on a 9-cm dish and incubated with Dulbecco’s modified Eagle medium (DMEM) (Invitrogen, Carlsbad, CA, USA) supplemented with 10% heat-inactivated fetal bovine serum (Sigma-Aldrich, St. Louis, MO, USA) at 37 °C overnight. Cells were transfected at < 50% confluency with each construct (10 μg/dish) mixed with Lipofectamine 2000 (Invitrogen, Carlsbad, CA, USA) in Opti-MEM I (Invitrogen). Media was changed after 4 h and cells were sampled two days later.

### 2.3. RNA Immunoprecipitation and Illumina Library Preparation

HeLa cells transfected with expression constructs were washed with phosphate-buffered saline (PBS) (−) five times. Total RNA purification and immunoprecipitation (IP) were performed with the RiboCluster Profiler RIP-Assay Kit #RN1001 (MBL International Corporation, Woburn, MA, USA). Immunoprecipitation was carried out using anti-GFP antibody (Clonetech Laboratories, Mountain View, CA, USA) or normal rabbit immunoglobulin G (IgG) as a control. Total RNA was purified with DNase (TURBO™ DNase (0.88 U) (Invitrogen)) and fragmented using mRNA-Seq Sample Prep Kit (Illumina, San Diego, CA, USA). RNA sized 200–400 nt was excised from the acrylamide gel and subject to library preparation. Cluster amplification and single-end sequencing was performed using the Illumina TruSeq SBS Kit v5-GA #FC-104-5001 and Illumina Genome Analyzer GAIIx according to the manufacturer’s protocol (read length: 36 nt). The sequence data was first converted to qseq format by CASAVA v1.8.2 (Illumina), and further converted to FASTQ format using the qseq2fastq converter provided by Kris Popendorf [12], and reads containing any base with a Phred quality score of less than 20 were filtered out using FASTX toolkit 0.0.13 [13] and custom python code to remove reads containing N’s and homo-polymers consisting solely of one type of nucleotide.

### 2.4. DEG Analysis (Tuxedo Pipeline)

The latest version of the human genome (hg38) was downloaded in FASTA format from gencode release 25. Since the reads were short (36 nt) and unstranded, we opted for mapping reads using tophat 2.0.9 [14,15] in combination with bowtie 1.0.0 [16], and allowed up to one mismatch per read while always favoring the best alignment. The gene transfer format (GTF) annotation files provided with gencode release 25 (all genes + tRNAs) were supplied as one file to the tophat input to limit the splicing junctions search to known splicing junctions. Furthermore, the coverage-search option was activated as recommended for short (<45 nt) reads, with alignments reported exclusively across “GT–AG” introns. All IP and input samples were processed similarly. The command line used for mapping was as follows:

tophat -G annotation.gtf --no-novel-juncs --no-novel-indels -N 1 --read-gap-length 0 --read-edit-dist 1 --read-realign-edit-distance 0 --bowtie1 -o ./tophat_output_samplename/ --coverage-search bowtie_index input.fastq

For differential expression analysis, the four “input” samples were used as input to cuffdiff from the cufflinks 2.1.1-4 package [14]. Equal dispersion and variance was assumed among all four samples with the ‘blind’ dispersion method. Note that cuffdiff automatically switches to ‘blind’ mode if only one replicate per sample is provided. This method is expected to give a conservative estimate of the number of significant differentially expressed genes (DEGs). Results were visualized using R 3.3.1 “Bug in Your Hair” [17], Bioconductor 3.3 [18], cummeRbund 2.14.0 [14], as well as custom code. The command line used for differential expression analysis was as follows, with C2, C4, C6, C7, representing ADAR1-p150, ADAR1-p110, ADAR2, and GFP input samples, respectively. The BAM alignment files (accepted_hits.bam) produced by the above tophat pipeline were used as input, requiring prior installation of samtools 0.1.19-1 [19].

cuffdiff -p 8 -o ./cuffdiff_output_folder -b genome_bowtie.fa -L C2,C4,C6,C7 -u annotation.gtf ./tophat_output_C2/accepted_hits.bam ./tophat_output_C4/accepted_hits.bam ./tophat_output_C6/accepted_hits.bam ./tophat_output_C7/accepted_hits.bam

### 2.5. Identification of Binding Targets (RIPSeeker)

RIPSeeker is an R package that was designed specifically to detect significantly enriched peaks in RIP-seq data [20]. Bin size was first optimized per chromosome by testing bins ranging from 200 to 400 bp in steps of 5. Chromosome X was not included in the initial test due to lack of memory (when not using a fixed bin size, RIPSeeker may use up to several hundred gigabytes of RAM and is unfortunately not optimized for multicore). The optimal bin size for all chromosomes tested was between 200 and 250. We subsequently used a fixed bin size of 200 bp for all chromosomes to optimize memory performance. For future reference, it took RIPSeeker 12 h on average to analyse one BAM file containing approximately 100 million alignments (multiple hits are included). To run RIPSeeker, the following R script was run from the terminal using a bash script, assuming that all necessary R packages (BSgenome.Hsapiens.UCSC.hg38, biomaRt, RIPSeeker, among others) are loaded:

# read path to BAM files

hg38<-getBSgenome("hg38",masked=F) #load human genome version 38

extdata.dir<-system.file("tophat_out", package="RIPSeeker") #set location of tophat output

bamFiles<-list.files(extdata.dir, "\\.bam$", recursive=T, full.names=T) #read filenames

outDir<-file.path("~/path/to/ripseeker_output") #set location of RIPSeeker output

file<-bamFiles[1] #set BAM file (replace 1 by number corresponding to desired file)

seqOut.file<-ripSeek(bamPath=file, genomeBuild="hg38", uniqueHit=T, assignMultihits=T, rerunWithDisambiguatedMultihits=T, binSize=200, biomart="ensembl", biomaRt_dataset="hsapiens_gene_ensembl", goAnno="org.Hs.eg.db", multicore=F, outDir=outDir) #multicore should always be set to FALSE

### 2.6. Downstream Functional Analysis of Results

For the classification of ADAR-bound transcripts, the Kyoto Encyclopedia of Genes and Genomes (KEGG) database [21] was used to identify possible functional pathways. PANTHER overrepresentation test was performed on Gene Ontology (GO) biological processes with default settings (Bonferroni correction for multiple testing, *p* < 0.05) with a tool from the GO Consortium [22,23].

## 3. Results

### 3.1. RNA Immunoprecipitation (RIP)-Sequencing Experimental Setup

**Figure 1:**
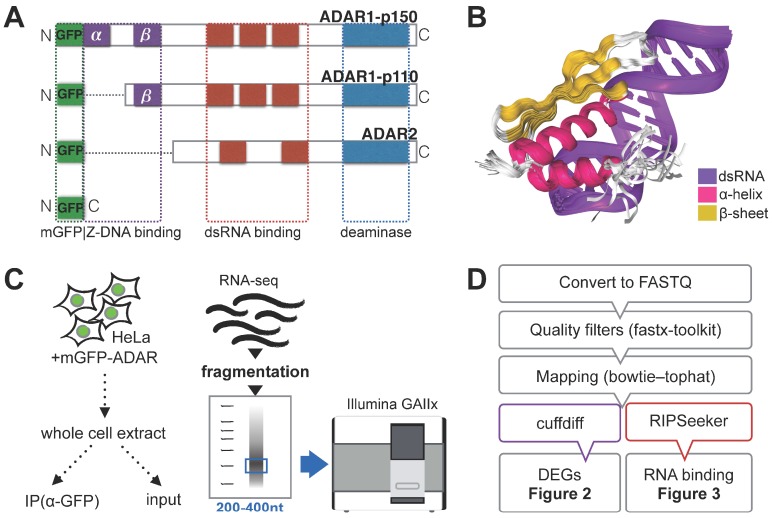
Experimental and computational workflow for RNA immunoprecipitation (RIP)-sequencing. (**A**) Schematic representation of known domains within adenosine deaminase acting on RNA (ADAR) enzyme isoforms with a catalytically-active deaminase domain. The final fusion proteins used in this experiment all harbor myc-green fluorescent protein (GFP) (mGFP) in the N-terminal region (left side in this figure). The dots within the protein sequence do not represent any real protein sequence, they were added in order to align and visually compare similar domains. Purple: Z-DNA binding domains; red: double-stranded RNA (dsRNA)-binding domains; blue: deaminase domain; green: mGFP; (**B**) the crystal structure of ADAR2 dsRNA-binding domain dsRBM1 bound to the free gluR-B R/G lower stem-loop (LSL) RNA rendered from PDB accession number (23LC). Different types of structures are represented in separate colors; (**C**) the experimental workflow for RIP-seq. HeLa cells are transfected with expression vectors for each mGFP-ADAR fusion protein. Part of the whole cell lysate was used for IP using anti-GFP antibody attached to magnetic beads, while the other part was used as a control (input). RNA was fragmented and material sized 200–400 nt was selected by gel electrophoresis and subjected to library preparation using mRNA-Seq Sample Prep Kit for Illumina GAIIx; and (**D**) the custom computational workflow used to obtain the results presented in this paper: differentially-expressed genes (DEGs) and ADAR-bound RNA targets.

Catalytically-active members of the ADAR family comprise three major isoforms, the functional domain organization of which are summarized in Figure 1A. Both the constitutive isoforms ADAR1-p110 and ADAR2 are mostly nuclear and undergo shuttling in and out of the nucleolus, while ADAR1-p110 also undergoes nucleocytoplasmic shuttling [24]. ADAR2 is the shortest and possesses two dsRNA-binding domains; the activity of its deaminase domain is thought to be less sequence-specific [25]. ADAR1-p110 possesses three dsRNA-binding domains and a Z-DNA binding domain (β). Interferon-induced ADAR1-p150 comprises of the sequence of ADAR1-p110 with an additional Z-DNA binding domain (α), which is known to localize to cytoplasmic stress granules [26]. Figure 1B illustrates the dsRNA-binding activity of ADARs by showing the crystal structure of ADAR2 dsRNA binding domain in complex with a dsRNA helix [27]. In order to identify differentially-expressed genes and preferentially-bound RNA species, each ADAR isoform was overexpressed in HeLa cells as a protein fusion construct with myc-GFP in the N-terminal region, and myc-GFP alone was in turn expressed as a control. RNA immunoprecipitation (RNA-IP or RIP) was carried out with a monoclonal antibody against GFP, and RNA from both the input and IP was size-selected on an acrylamide gel for Illumina^®^ library construction. For the study of mRNAs and long non-coding RNAs, total RNA was fragmented and fragments sized 200-400 nt were purified and subjected to next-generation sequencing by GAIIx (Figure 1C, Materials and Methods). In total, seven datasets were generated: GFP input, ADAR1-p110 IP and input, ADAR2 IP and input, as well as ADAR1-p150 IP and input. Each dataset was analyzed according to the computational workflow presented in Figure 1D. First, raw reads were pre-processed by converting to the standard FASTQ format and filtering out low-quality reads and artefacts. The quality filtering step yielded 22 to 30 million reads per sample, which were then mapped to the human genome. Differentially-expressed genes (DEGs) were identified using the tuxedo pipeline (tophat–cuffdiff) [14,15] optimized for short unstranded reads (Material and Methods) [28]. Finally, RNA binding targets were identified using RIPseeker, which uses hidden Markov models to accurately identify enriched transcripts from RIP-seq alignment files [20].

### 3.2. Effect of ADAR Isoform Overexpression on Global Gene Expression

**Figure 2:**
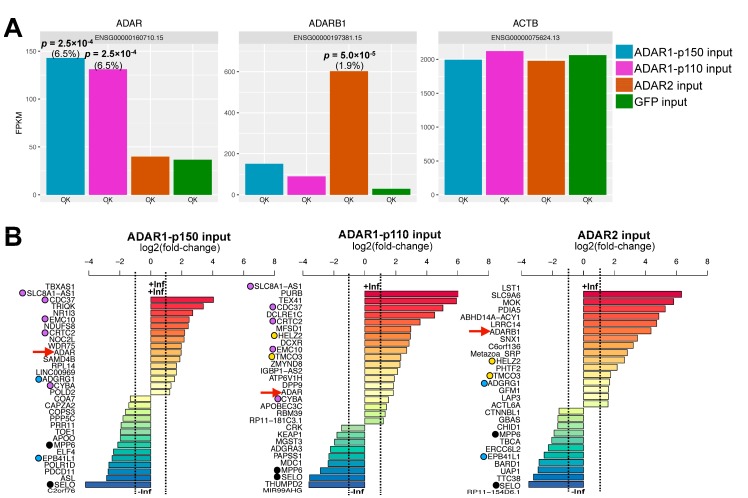
Differentially expressed genes upon specific ADAR isoform overexpression. (**A**) Coverage of the *ADAR* (*ADAR1*), *ADARB1* (*ADAR2*), and *ACTB* genes expressed in reads per kilobase per million reads (RPKM). ADAR codes for both the ADAR1-p150 and ADAR1-p110 isoforms, ADARB1 codes for ADAR2 isoform, and ACTB codes for Actin B. The *p*-value relative to GFP input is indicated in bold and the corresponding false discovery rate (FDR) in brackets; (**B**) Fold-change relative to GFP input for significant DEGs (FDR ≤ 6.5%; *p* ≤ 2.5 × 10^−4^) expressed on a logarithmic scale. The *ADAR* and *ADARB1* controls are highlighted with a red arrow. Black filled circles: DEGs common to all three ADAR isoforms; purple filled circles: common to ADAR1-p150 and ADAR1-p110; blue filled circles: common to ADAR1-p150 and ADAR2; yellow filled circles: common to ADAR1-p110 and ADAR2.

DEGs were identified by the expression levels in each ADAR input sample relative to the GFP input sample. To this end, the standard tuxedo pipeline described by Trapnell et al. [14] was adapted using available parameters most suitable for short unstranded reads (Materials and Methods). Second, the cuffdiff implementation for identifying DEGs goes beyond a traditional Poisson model for RNA-seq by providing several methods to estimate the dispersion present in a group of replicates. For instance, it provides a useful option for dispersion estimation in the case of only one replicate per sample, as is the case here. As shown in Supplementary Figure S1, the distribution of read counts and dispersion was fairly similar among samples. Therefore, we assumed equal variance among all samples, and treated all samples as replicates of a single condition. This trick is expected to produce a rather conservative estimation of DEGs [28]. The final output contained the level of significance and false discovery rate (FDR) for each gene. The overexpression of ADAR1-p150/-p110, and ADAR2 was successfully detected in each sample, respectively, with FDRs of 6.5%, 6.5%, and 1.9%, respectively, and levels of significance lower than 2.5 × 10^−4^ (Figure 2A). It has to be noted that, since DEG analysis reports gene expression changes per gene unit, it groups together the expression of isoforms originating from the same locus, such as ADAR1-p110 and –p150. Based on these results, DEGs that were up- or down-regulated more than two-fold relative to GFP input were kept only if their FDR and *p*-values were below the 6.5% and 2.5 × 10^−4^, respectively (Figure 2B). Detailed fold-change values and the gene description for each DEG are presented in Supplementary Table S1.

MPP6 and SELO transcripts were downregulated in all cases, meaning they are likely nonspecific. Few DEGs were in common between samples overexpressing different ADAR isoforms, except for ADAR1-p150 and ADAR1-p110, which both resulted in the significant upregulation of five common genes. Since ADAR1-p150 consists of the full amino acid sequence of ADAR1-p110 with an extension of N-terminal 295 amino acids [29], some overlap in function is to be expected. This included the up-regulation of two major phosphoinositide 3-kinases (PI3K)-Akt signaling pathway elements, cell division cycle 37 (CDC37) and mammalian target of rapamycin (mTOR)C2 (CRTC2), which are known to activate the RAC-alpha serine/threonine-protein kinase AKT by phosphorylation [30]. This pathway is overactive in a multitude of cancers, suggesting a tumorigenic role of excess ADAR1 expression. Consistent with this observation, strong inhibition of the mir-99a-let-7c cluster host gene MIR99AHG guiding the expression of let-7c, miR-99a and miR-125b was also observed in the ADAR1-p110 input sample compared to GFP input (Figure 2B, middle). Downregulation of this miRNA cluster was recently shown to induce tumorigenesis due to a loss of inhibition of key inflammatory cytokines involved in the IL-6/signal transducer and activator 3 (STAT3) pathway in cholangiocarcinoma [31]. An excess of ADAR1-p110 was also shown to be tumorigenic in the case of lung cancer [32]. The effect of different ADAR isoforms on miRNA maturation and expression levels remains to be investigated in an isoform-specific manner, and will be the subject of further study. Interestingly, the overexpression of ADAR1-p110 resulted in the up-regulation of another deaminase, APOBEC3C, which is localized in the nucleus and speculated to promote dC-to-dU DNA (and possibly also C-to-U RNA) editing [33].

In contrast, the genes influenced by *ADAR2* overexpression were less well documented. We observed up-regulation of MOK mitogen-activated protein (MAP)-kinase, and differential expression of proteins involved in vesicular and intracellular trafficking (SLC9A6, SNX1, GBAS) and metabolic enzymes (LAP3, UAP1). Notably, *ADAR2* overexpression led to the down-regulation of spliceosomal core component CTNNBL1, consistent with a recent report showing a negative correlation between splicing and editing by *ADAR2* [34]. Finally, we also observed down-regulation of DNA repair factors ERCC6L2 (Snf2 family of helicase-like proteins) and BRCA1-associated RING domain 1 (BARD1), a factor involved in the early steps of homologous recombination. Therefore, *ADAR2* overexpression may also affect the ability of cells to repair DNA. 

### 3.3. ADAR Isoforms Bind to Distinct Targets Genome-Wide

RIP-seq analysis is typically carried out by simply comparing the coverage in the IP fraction with that of input samples, which gives an idea of the relative enrichment without providing confidence estimates. Quantification remains difficult for genomes containing a high number of repeats, as reads mapping to those regions may not be assigned accurately, and IP and input samples are not always directly comparable due to potentially divergent coverage of these repeat regions between IP and input. One solution is to perform peak calling on the IP sample, but many peak callers were designed for chromatin immunoprecipitation (ChIP-seq) data and, therefore, assume the presence of tandem peaks. RIPSeeker was designed specifically for RIP-seq, and uses machine learning on an alignment file to first model peak enrichment within the IP sample while taking into account only unique hits. Then, it reassigns multiple hits to their most likely location based on posterior probability, according to a “rich gets richer” model. Peak calling is then performed again on the unique and disambiguated multihits. Although computationally intensive, this method performs extremely well compared to other methods even when given an IP sample alignment file as sole input [20]. The parameters were optimized as described in Materials and Methods.

**Figure 3:**
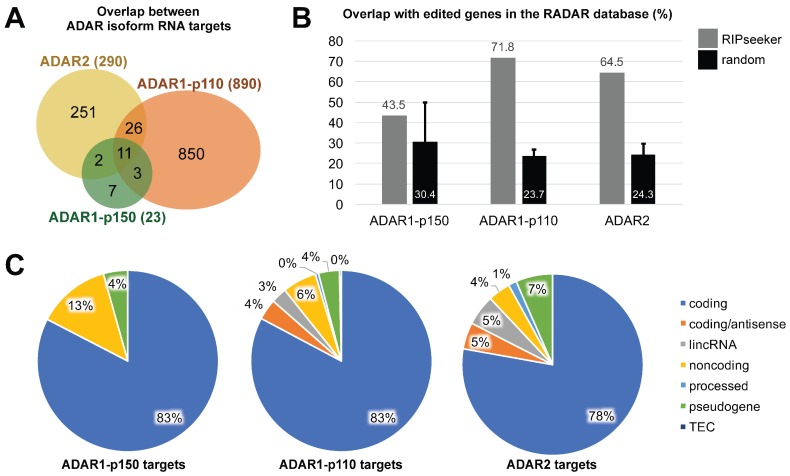
Identification of ADAR isoform-specific binding targets by RIPSeeker. (**A**) Venn diagram showing the overlap between the unique gene IDs of ADAR1-p150-, ADAR1-p110- and ADAR2-bound transcripts. (**B**) Overlap between unique gene IDs for each ADAR and unique gene IDs registered in the RADAR A-to-I editing site database (grey), compared to the mean overlap of 10,000 datasets chosen randomly from the human gene annotation file. Each random data set was similar in size to the corresponding ADAR; and (**C**) pie charts representing the proportion of RNA categories found in each ADAR-bound sample, respectively. Blue: coding transcripts; other colors: non-coding or uncharacterized transcripts.

Peak calling with RIPSeeker was performed on each IP alignment file, which produced a list of peaks and their corresponding candidate bound transcripts for each ADAR isoform (Supplementary Table S2). Although RIPSeeker outputs neighboring gene features in the case a peak does not completely overlap with a gene annotation, we filtered these out and only kept peaks that were fully included inside a given annotation. This yielded 23, 890, and 290 unique gene identifiers (IDs) for candidate transcripts bound by ADAR1-p150, ADAR1-p110, and ADAR2, respectively. Although ADAR1-p150 bound relatively fewer targets, most of which were also bound by other ADAR isoforms, the general overlap was minimal, with only 11 transcripts out of 1144 (less than 1% of all candidates) bound by all three ADARs (Figure 3A). ADAR1-p110 and ADAR2 targets were especially clearly divided. 

ADAR binding does not necessarily entail A-to-I editing, but one may expect that ADAR-bound transcripts are more frequently edited than unbound transcripts. Indeed, as shown in Figure 3B, the transcripts detected in our study highly overlapped with those registered in the Rigorously Annotated Database of A-to-I RNA editing (RADAR) database (v2), that lists previously published A-to-I editing sites [35]. However, since this database currently lists more than 20,000 unique gene IDs, and the human genome annotation file contains a little above 60,000 entries, we needed to make sure that this high overlap was not merely due to chance. This was tested by generating 10,000 sets of gene IDs picked randomly from the gene annotation file, and calculating the percentage of overlap with transcripts in the RADAR database for each random set. The mean and standard deviation of this bootstrapping are presented as black bars in Figure 3B. Overall, these results show that the ADAR1-p110 and ADAR2-bound targets identified by this study are significantly enriched in edited transcripts.

Finally, the candidate ADAR-bound transcripts were manually curated into the following gene type categories based on information available on the Ensembl website: (1) coding, for protein-coding; (2) coding/antisense, when RIPSeeker could not determine which strand was relevant and both a coding transcript and its overlapping antisense RNA were detected ; (3) lincRNA, for long intergenic non-coding RNAs ; (4) non-coding, for any other type of long non-coding RNA, most of which were antisense RNAs; (5) processed, for transcripts indicated as such in the Ensembl database; (6) pseudogene, including known and unprocessed pseudogenes ; (7) TEC, for “to be experimentally confirmed”, again when indicated as such (Figure 3C). Notably, ADAR1-p110 non-coding targets included small nucleolar RNA C/D box 3C (SNORD3C) and the miRNA precursor MIR568, which are both associated with the long non-coding RNA class (4). ADAR2 bound to a slightly higher proportion of non-coding transcripts than ADAR1-p110, although the difference was not quite significant (2 × 2 contingency table Fisher’s exact test two-tailed *p*-value: 0.0738). When interpreting these results, one should keep in mind that coding transcripts might be overrepresented, as the genome annotation file available at GENCODE does not contain annotations for repeat elements such as Alu, short interspersed nuclear elements (SINEs), or long interspersed elements (LINEs). The proportion of non-coding transcripts bound by ADARs is expected to be much higher in reality than represented in Figure 3C, as Alu elements are heavily targeted by ADARs [36]. The question is open whether the reassignment of reads with multiple hits by RIPSeeker would be suitable for heavily repeated sequences, since there would be few unique reads available to perform the initial peak modeling step on those regions.

KEGG pathway analysis seem to indicate that ADAR1-p110 binds a great number of transcripts involved in Pathways in Cancer (ko05200), to which 14 genes corresponded out of the 159 ADAR1-p110-bound candidates currently registered (Table 1). The KEGG pathway search did not yield any convincing results for the transcripts bound by either of the other two ADAR isoforms, probably due to poor overlap with KEGG-registered genes. To increase functional prediction efficacy, GO enrichment for biological processes was performed using default parameters (Materials and Methods), and statistically significant results with an enrichment of more than two-fold relative to the expected number of genes are summarized in Table 2. Nothing statistically significant was found for ADAR2-bound targets, but both ADAR1 isoforms seemed to target more transcripts involved in the regulation of translation, mRNA degradation, and viral metabolism.

## 4. Discussion

Enzyme isoforms in higher eukaryotes tend to specialize in their function, yet they can be difficult to distinguish experimentally, especially when these isoforms derive from alternative splicing. For instance, one can design ADAR1-p150-specific antibodies, but antibodies targeting ADAR1-p110 will inevitably target both ADAR1-p110 and -p150. This study attempts to address this issue of ADAR isoform specificity by overexpressing each major ADAR isoform with a GFP-tag in the N-terminal. Although the overexpression of a given gene may have secondary effects on global gene expression, at least the global distribution of gene expression values (RPKM) did not significantly change between samples (ADAR1-p150, -110, ADAR2, GFP inputs) (Supplementary Figure S1). One other issue that is specific to RNA editing is to preserve the ability to detect RNA editing sites on target transcripts. Other studies have tried crosslinking immunoprecipitation (CLIP)-seq, a method that stabilizes the interaction between a protein and its target RNA by UV-crosslinking [37]. Although this prevents the dissociation and re-association of ADAR on its target RNA, this introduces sequencing errors at the binding site due to irreversible effects of crosslinking, so that CLIP-seq can identify binding but not editing. Furthermore, traditional UV crosslinking is notably inefficient in the case of extended dsRNA targets [10], as is the case for ADARs, meaning that current CLIP-seq methods may introduce a bias for RNA targets containing more bulges and loops. One of the main goals of this paper was to present a crosslinking-free alternative by applying RIP-seq to the detection of ADAR-bound transcripts. The fact that we got a very significant overlap between ADAR-bound targets and transcripts registered in the major A-to-I editing database suggests that crosslinking is not absolutely necessary for the study of ADAR targets. The absence of crosslinking makes it theoretically possible to detect actual RNA editing sites within the IP samples. However, our current study was tentatively done on unstranded short reads from total RNA, which is not optimal for downstream detection of RNA editing sites. To this end, we would like to recommend (1) filtering out ribosomal RNA to maximize coverage; and (2) using strand-specific data with a longer read length to enable the study of bidirectional loci, as well as enhance signal-to-background ratio when detecting editing sites. Another challenge that arose was that the small number of candidate targets bound by ADAR1-p150 compared to other ADARs. Although this might reflect biological function, because ADAR1-p150 is the longest isoform, we speculate that the addition of a myc-GFP tag resulted in a protein so large that it may not have been overexpressed as efficiently as the other ADARs. We plan to address these issues in the near future. Meanwhile, the RIP-seq method presented here may be readily applied to other cell lines and various populations RNA may be enriched for the targeted study of longer mRNAs and lncRNAs, or smaller RNAs such as miRNAs.

Previously, ADAR1 overexpression was shown to have an inhibitory effect on iPS cell (iPSC) reprogramming, and the expression of ADAR1 in human embryonic stem cells (hESCs) resulted in the induction of differentiation-related genes [38]. Another study was unable to achieve overexpression of ADAR1-p110 in hESCs by traditional methods, suggesting that ADAR1 expression is tightly regulated in development [39]. Furthermore, it was also reported that iPSCs derived from cells in which ADAR1 was down-regulated exhibited the characteristics of cancer cells shortly after iPSC colony formation [38]. A-to-I editing is also reported to be altered in several cancers [31,39], and the results presented here are consistent with other studies suggesting a role of ADAR1 in cancer formation [40]. Indeed, the overexpression of ADAR1-p110 resulted in the up-regulation of CDC37 and mTORC2, which are involved in the PI3K-Akt signaling pathway (Figure 2C, Table 1). Furthermore, RIP-seq analysis revealed the binding by ADAR1-p110 of many transcripts involved in cancer, including major players such as β-catenin, transforming growth factor-beta (TGF-β) receptor, Raf, Rho, and nuclear factor-kappa B (NFκB) (Table 2). Further experimental validation is expected to confirm the molecular mechanism of ADAR oncogenicity.

## 5. Conclusions

This study is the first to present RIP-seq as a method to analyze the target specificity of all three major ADAR isoforms. We found that the overexpression of each ADAR isoform induces differential expression of distinct sets of genes, and that the genome-wide binding preferences of each isoform are clearly distinct, and in particular hint towards the mechanism of ADAR1-p110 in tumorigenesis. Furthermore, ADAR-bound targets substantially overlapped with transcripts for which at least one editing site is registered in the database of A-to-I editing sites. This shows that, contrary to current methods, such as CLIP-seq, RIP-seq may be more suitable for downstream detection of editing sites due to the absence of crosslinking.

## Figures and Tables

**Table 1:** ADAR1-p110-bound transcripts present in KEGG Pathways in Cancer (ko05200).

Gene ID	KEGG Name	KEGG ID	Description
*APPL1*	APPL	K08733	DCC-interacting protein 13 alpha
*CTNNB1*	β-catenin	K02105	Catenin beta 1
*RHOA*	Rho, Rac/Rho	K04513	Ras homolog gene family, member A
*GSK3B*	GSK-3β	K03083	Glycogen synthase kinase 3 beta
*ITGB1*	ITGB	K05719	integrin beta 1
*GNB1*	βγ	K04536	Guanine nucleotide-binding protein G(I)/G(S)/G(T) subunit beta-1
*VHL*	VHL	K03871	von Hippel-Lindau disease tumor suppressor
*MLH1*	hMLH1	K08734	DNA mismatch repair protein MutL homolog 1
*TGFBR2*	TGFβRII	K04388	Transforming growth factor (TGF)-beta receptor type 2
*MITF*	MITF	K09455	Melanogenesis associated transcription factor
*RAF1*	Raf	K04366	RAF proto-oncogene serine/threonine-protein kinase
*TFG*	TRK	K09292	Tyrosine kinase receptor (TRK)-fused gene
*NCOA4*	RET/PTC	K09289	Nuclear receptor coactivator 4
*NFKB1*	NFκB	K02580	Nuclear factor NF-kappa-B p105 subunit

**Table 2:** Positively enriched gene ontology (GO) biological processes for ADAR-bound transcripts.

Bound Isoform	GO Biological Process	Hits	Expected	Fold-Enrichment	*p*-Value
**ADAR1-p150**	SRP-dependent cotranslational protein targeting to membrane	4	0.09	45.99	1.34 × 10^−2^
	Viral transcription	4	0.1	38.39	2.73 × 10^−2^
	Nuclear-transcribed mRNA catabolic process, nonsense-mediated decay	4	0.11	36.79	3.23 × 10^−2^
	rRNA processing	5	0.24	20.91	2.64 × 10^−2^
**ADAR1-p110**	Nuclear-transcribed mRNA catabolic process	25	6.95	3.6	6.34 × 10^−2^
	SRP-dependent cotranslational protein targeting to membrane	15	3.39	4.43	2.21 × 10^−2^
	Viral life cycle	33	10.59	3.12	1.63 × 10^−4^
	Translation	37	16.09	2.3	3.47 × 10^−2^
**ADAR2**	No significantly enriched GO biological process				

## Supplementary Materials

The following are available online at www.mdpi.com/2073-4425/8/2/68/s1, Figure S1: Statistics on the RNA-seq data sets for differential expression analysis, Table S1: List of differentially expressed genes, Table S2: List of ADAR-bound candidates detected by RIPSeeker.

## References

[1] Higuchi M., Single F.N., Köhler M., Sommer B., Sprengel R., Seeburg P.H. (1993). RNA editing of AMPA receptor subunit GluR-B: A base-paired intron-exon structure determines position and efficiency. Cell.

[2] Chen C.X., Cho D.S., Wang Q., Lai F., Carter K.C., Nishikura K. (2000). A third member of the RNA-specific adenosine deaminase gene family, ADAR3, contains both single- and double-stranded RNA binding domains. RNA.

[3] Wang Q., Miyakoda M., Yang W., Khillan J., Stachura D.L., Weiss M.J., Nishikura K. (2004). Stress-induced apoptosis associated with null mutation of *ADAR1* RNA editing deaminase gene. J. Biol. Chem..

[4] Higuchi M., Maas S., Single F.N., Hartner J., Rozov A., Burnashev N., Feldmeyer D., Sprengel R., Seeburg P.H. (2000). Point mutation in an AMPA receptor gene rescues lethality in mice deficient in the RNA-editing enzyme ADAR2. Nature.

[5] Patterson J.B., Thomis D.C., Hans S.L., Samuel C.E. (1995). Mechanism of interferon action: double-stranded RNA-specific adenosine deaminase from human cells is inducible by alpha and gamma interferons. Virology.

[6] Crick F.H. (1966). Codon-anticodon pairing: The wobble hypothesis. J. Mol. Biol..

[7] Li J.B., Levanon E.Y., Yoon J.-K., Aach J., Xie B., Leproust E., Zhang K., Gao Y., Church G.M. (2009). Genome-wide identification of human RNA editing sites by parallel DNA capturing and sequencing. Science.

[8] Nishikura K. (2010). Functions and regulation of RNA editing by ADAR deaminases. Annu. Rev. Biochem..

[9] George C.X., Samuel C.E. (1999). Human RNA-specific adenosine deaminase ADAR1 transcripts possess alternative exon 1 structures that initiate from different promoters, one constitutively active and the other interferon inducible. Proc. Natl. Acad. Sci. USA.

[10] Liu Z.R., Wilkie A.M., Clemens M.J., Smith C.W. (1996). Detection of double-stranded RNA-protein interactions by methylene blue-mediated photo-crosslinking. RNA.

[11] Nishi K., Nishi A., Nagasawa T., Ui-Tei K. (2013). Human TNRC6A is an Argonaute-navigator protein for microRNA-mediated gene silencing in the nucleus. RNA.

[12] Popendorf K. qseq2fastq. www.dna.bio.keio.ac.jp/~krisp/qseq2fastq/.

[13] Hannon laboratory. http://hannonlab.cshl.edu/fastx_toolkit/.

[14] Trapnell C., Roberts A., Goff L., Pertea G., Kim D., Kelly D.R., Pimentel H., Salzberg S.L., Rinn J.L., Pachter L. (2012). Differential gene and transcript expression analysis of RNA-seq experiments with TopHat and Cufflinks. Nat. Protoc..

[15] Kim D., Pertea G., Trapnell C., Pimentel H., Kelley R., Salzberg S. (2013). TopHat2: Accurate alignment of transcriptomes in the presence of insertions, deletions and gene fusions. Genome Biol..

[16] Langmead B., Trapnell C., Pop M., Salzberg S.L. (2009). Ultrafast and memory-efficient alignment of short DNA sequences to the human genome. Genome Biol..

[17] R Development Core Team (2008). R: A Language and Environment for Statistical Computing.

[18] Huber W., Carey V.J., Gentleman R., Anders S., Carlson M., Carvalho B.S., Bravo H.C., Davis S., Gatto L., Girke T. (2015). Orchestrating high-throughput genomic analysis with Bioconductor. Nat. Methods.

[19] Li H., Handsaker B., Wysoker A., Fennell T., Ruan J., Homer N., Marth G., Abecasis G., Durbin R., 1000 Genome Project Data Processing Subgroup (2009). The Sequence Alignment/Map format and SAMtools. Bioinformatics.

[20] Li Y., Zhao D.Y., Greenblatt J.F., Zhang Z. (2013). RIPSeeker: A statistical package for identifying protein-associated transcripts for RIP-seq experiments. Nucleic Acids Res..

[21] Kanehisa M., Sato Y., Kawashima M., Furumichi M., Tanabe M. (2016). KEGG as a reference resource for gene and protein annotation. Nucleic Acids Res..

[22] Thomas P.D., Campbell M.J., Kejariwal A., Mi H., Karlak B., Daverman R., Diemer K., Muruganujan A., Narechania A. (2003). PANTHER: A library of protein families and subfamilies indexed by function. Genome Res..

[23] Mi H., Dong Q., Muruganujan A., Gaudet P., Lewis S., Thomas P.D. (2010). PANTHER version 7: Improved phylogenetic trees, orthologs and collaboration with the Gene Ontology Consortium. Nucleic Acids Res..

[24] Desterro J.M.P., Keegan L.P., Lafarga M., Berciano M.T., O’Connell M., Carmo-Fonseca M. (2003). Dynamic association of RNA-editing enzymes with the nucleolus. J. Cell Sci..

[25] Barraud P., Allain F.H.-T. (2012). ADAR proteins: Double-stranded RNA and Z-DNA binding domains. Curr. Top. Microbiol. Immunol..

[26] Ng S.K., Weissbach R., Ronson G.E., Schadden A.D. (2013). Proteins that contain a functional Z-DNA-binding domain localize to cytoplasmic stress granules. Nucleic Acids Res..

[27] Schwartz T., Behike J., Lowenhaupt K., Heinemann U., Rich A. (2001). Structure of the DLM-1-Z-DNA complex reveals a conserved family of Z-DNA-binding proteins. Nat. Struct. Biol..

[28] Cufflinks Manual. http://cole-trapnell-lab.github.io/cufflinks/cuffdiff/#cross-replicate-dispersion-estimation-methods.

[29] Samuel C.E. (2011). Adenosine deaminases acting on RNA (ADARs) are both antiviral and proviral dependent upon the virus. Virology.

[30] King D., Yeomanson D., Bryant H.E. (2015). PI3King the Lock: Targeting the PI3K/Akt/mTOR Pathway as a Novel Therapeutic Strategy in Neuroblastoma. J. Pediatr. Hematol. Oncol..

[31] Lin K.-Y., Ye H., Han B.-W., Wang W.-T., Wei P.-P., He B., Li X.-J., Chen Y.-Q. (2016). Genome-wide screen identified let-7c/miR-99a/miR-125b regulating tumor progression and stem-like properties in cholangiocarcinoma. Oncogene.

[32] Anadón C., Guil S., Simó-Riudalbas L., Moutinho C., Setien F., Martínez-Cardús A., Moran S., Villanueva A., Calaf M., Vidal A. (2016). Gene amplification-associated overexpression of the RNA editing enzyme ADAR1 enhances human lung tumorigenesis. Oncogene.

[33] Jarmuz A., Chester A., Bayliss J., Gisbourne J., Dunham I., Scott J., Navaratnam N. (2002). An anthropoid-specific locus of orphan C to U RNA-editing enzymes on chromosome 22. Genomics.

[34] Licht K., Kapoor U., Mayrhofer E., Jantsch M.F. (2016). Adenosine to inosine editing frequency controlled by splicing efficiency. Nucleic Acids Res..

[35] Ramaswami G., Li J.B. (2014). RADAR: A rigorously annotated database of A-to-I editing. Nucleic Acids Res..

[36] Kim D.D.Y., Kim T.T.Y., Walsh T., Kobayashi Y., Matise T.C., Byske S., Gabriel A. (2004). Widespread RNA editing of embedded alu elements in the human transcriptome. Genome Res..

[37] Bahn J.H., Ahn J., Lin X., Zhang Q., Lee J.H., Civelek M., Xiao X. (2015). Genomic analysis of ADAR1 binding and its involvement in multiple RNA processing pathways. Nat. Commun..

[38] Germanguz I., Shtrichman R., Osenberg S., Ziskind A., Novak A., Domev H., Laevsky I., Jacob-Hirsch J., Feiler Y., Rechavi G. (2014). ADAR1 is involved in the regulation of reprogramming human fibroblasts to induced pluripotent stem cells. Stem Cells Dev..

[39] Shtrichman R., Germanguz I., Mandel R., Ziskind A., Nahor I., Safran M., Osenberg S., Sherf O., Rechavi G., Itskovitz-Eldor J. (2012). Altered A-to-I RNA editing in human embryogenesis. PLoS ONE.

[40] Han L., Diao L., Yu S., Xu X., Li J., Zhang R., Yang Y., Werner H.M., Eterovic A.K., Yuan Y. (2015). The Genomic Landscape and Clinical Relevance of A-to-I RNA Editing in Human Cancers. Cancer Cell.

