# Supplementary material for: Differential Binding of Three Major Human ADAR Isoforms to Coding and Long Non-Coding Transcripts
Source: Genes (Basel). 2017 Feb 11;8(2):68. doi: 10.3390/genes8020068 (PMC5333057; doi:10.3390/genes8020068)
Supplement: Supplementary file 1 [file genes-08-00068-s001.zip › genes-172915 sups/genes-Fig-S1.pdf]

Supplementary Materials: Differential Binding of Three Major Human ADAR Isoforms to Coding and Long Non-Coding Transcripts

Josephine Galipon, Rintaro Ishii, Yutaka Suzuki, Masaru Tomita and Kumiko Ui-Tei

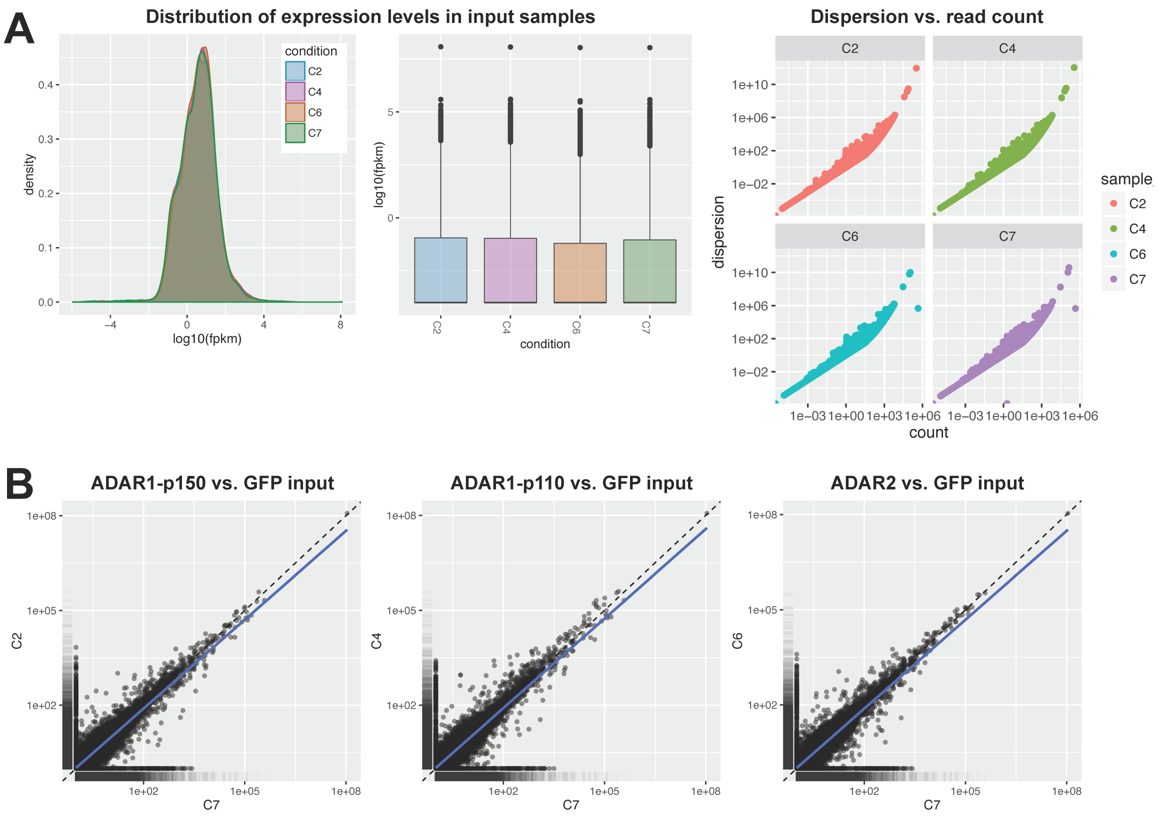

Figure S1. Statistics on the RNA-seq data sets for differential expression analysis. (**A**) Left: density distribution of the expression levels of transcripts in each sample on a log10 scale. Middle: box plot representing the distribution of expression levels in each sample. Right: scatter plot representing the dispersion as a function of read count for each sample; (**B**) Scatter plots of the expression levels in the input sample of each ADAR isoform relative to GFP input. C2, C4, C6, and C7 stand for ADAR1-p150, ADAR1-p110, ADAR2, and green fluorescent protein (GFP), respectively.
